# Reliability and Validity of the Simple Qi Deficiency Score in Patients with Post-COVID-19 Condition

**DOI:** 10.3390/medicina62071421

**Published:** 2026-07-22

**Authors:** Kazuki Tokumasu, Yoshifumi Sugiyama, Yuki Otsuka, Yohei Masuda, Nobuyoshi Matsuki, Keigo Ueda, Fumio Otsuka

**Affiliations:** 1 Department of General Medicine, Graduate School of Medicine, Dentistry and Pharmaceutical Sciences, Okayama University, 2-5-1 Shikata-cho, Kita-ku, Okayama 700-8558, Japan; otsuka@s.okayama-u.ac.jp (Y.O.); fumiotsu@md.okayama-u.ac.jp (F.O.); 2 Division of Clinical Epidemiology, Research Centre for Medical Science, The Jikei University School of Medicine, Tokyo 105-8461, Japan; 3 Department of Epidemiology, Medical School, Okayama University, Okayama 700-8530, Japan; 4 Clinical & Educational Centre for Kampo Medicine, Okayama University Hospital, Okayama 700-8558, Japan

**Keywords:** simple Qi deficiency scale, Qi deficiency, general fatigue, Japanese traditional (Kampo) medicine, long COVID, Post-COVID-19 condition

## Abstract

*Background and Objectives:* Persistent COVID-19 sequelae, often referred to as “long COVID”, still constitute a major global medical challenge. In particular, general fatigue is the most widely reported, serious symptom. In the framework of Japanese traditional (Kampo) medicine (JTM), post-infection fatigue is frequently considered a “Qi deficiency”, a significant loss of vital energy. In JTM, accurately diagnosing Qi deficiency is essential for determining the appropriate treatment. However, one challenge in JTM diagnosis is that mastering traditional diagnostic techniques takes time. We sought to validate the “Qi deficiency score”, a quantitative patient-reported outcome measure designed to assess Qi deficiency in patients suffering from long COVID. *Materials and Methods:* This was a methodological study. We conducted a cross-sectional study of 237 patients who sought treatment at the post-COVID-19 clinic (COVID-19 Aftercare Clinic) at Okayama University Hospital. The patient population was randomly split into two cohorts: one for Exploratory Factor Analysis (EFA, *n* = 122) and another for Confirmatory Factor Analysis (CFA, *n* = 115). The simple Qi-deficiency score comprises eight items derived from Terasawa’s diagnostic criteria, with a total range of 0–56. We evaluated internal consistency using Cronbach’s α/McDonald’s ω and examined structural validity. Convergent validity was established by examining correlations between the simple Qi deficiency score and the Fatigue Assessment Scale (FAS), the Self-rating Depression Scale (SDS), and Quality of Life (QOL) metric. *Results:* The score’s internal consistency (α = 0.64 and ω = 0.63) was considered modest for screening traditional clinical syndromes. EFA showed major factors representing Qi deficiency symptoms. CFA supported a one-factor model with modest fit indices (CFI = 0.87; RMSEA = 0.079; SRMR = 0.077; TLI = 0.82). Factor loadings revealed that “Body feels tired (now)” (0.98) and “Tires easily (tendency)” (0.73) were the most significant symptoms. Convergent validity showed a strong positive correlation with the FAS (*r* = 0.61, ρ = 0.60) and a moderate correlation with the SDS (*r* = 0.55, ρ = 0.55). A significant negative correlation was found with QOL scores (*r* = −0.42, ρ = −0.43). *Conclusions*: The simple Qi deficiency score may be useful for evaluating Qi deficiency in patients with post-COVID-19 condition.

## 1. Introduction

Although the COVID-19 pandemic has passed, global healthcare systems continue to treat many patients with long-term sequelae of the virus, widely known as long COVID or post-COVID-19 condition (PCC) [1,2,3]. The prevalence of these sequelae was >20% of infected individuals from a systematic review and meta-analysis [4]. General fatigue is consistently reported as the most frequent and serious sequelae, affecting approximately 28.4–34.8% of patients [4]. This fatigue often proves resistant to conventional Western therapies, persisting for months or even years and severely impairing daily function [5,6,7].

Japanese traditional (Kampo) medicine (JTM) is often used for management of post-infectious syndromes [8]. JTM approaches the condition as a systemic imbalance rather than targeting a specific pathogen. The chronic exhaustion seen in PCC is viewed as a manifestation of “Qi deficiency” in JTM. Qi deficiency expresses a lack of vital energy required for physiological processes. This presents not only as fatigue, but also includes symptoms such as lack of motivation, daytime drowsiness, loss of appetite, and increased proneness to diarrhea [9].

“Hochuekkito” (a formula literally meaning “to supplement the middle heater and boost Qi” which is also known as “Bu Zhong Yi Qi Tang” in traditional Chinese medicine) has become the most frequently prescribed Kampo formulation for long COVID in Japan [8]. While clinical experience and pilot studies suggest its efficacy [10], evaluation methods for patients with Qi-deficiency must assess effectiveness of therapeutic interventions.

The gold standard for diagnosing “Qi Deficiency” was originally developed by Katsutoshi Terasawa [11]. However, these criteria rely heavily on physical examination skills unique to JTM specialists, such as pulse diagnosis (assessing pulse strength) and abdominal palpation (checking for abdominal wall flaccidity) [12]. The problem is that such findings are subjective and difficult for general practitioners.

General fatigue in patients with PCC and long COVID has often been evaluated using the Fatigue Assessment Scale (FAS) [13] and the Chalder Fatigue Questionnaire [7]. Because the FAS has also been validated for assessment of fatigue in long COVID, it is considered valid and useful [13]. While there are general scales for measuring fatigue, no indicators based on the concept of Qi deficiency in JTM have been validated. To address this need, we developed the “simple Qi deficiency score” by extracting only subjective symptoms from Terasawa’s criteria to create a patient-reported outcome measure. While promising, psychometric properties of this simplified tool have not yet been rigorously tested in the context of long COVID. We assessed the reliability and validity of the simple Qi deficiency score in a cohort of Japanese patients with long COVID.

## 2. Methods

### 2.1. Research Design

We conducted this cross-sectional study at the Department of General Medicine, Okayama University Hospital. To treat patients with lingering symptoms, the hospital established a dedicated COVID-19 Aftercare Clinic (CAC) in February 2021. Patients are referred to this clinic if their symptoms persist for more than four weeks following infection.

### 2.2. Participants

We reviewed clinical records of patients visiting the CAC between 15 February 2021 and 25 June 2024. Adults (aged >18) who answered all questions needed to calculate the simple Qi deficiency score during their initial evaluation were included. No exclusion criteria were applied in this validation study. However, two cases were excluded due to missing responses to the questionnaire. In total, 237 patients were included. To avoid overfitting, we randomly split this sample into two groups: one for Exploratory Factor Analysis (EFA, *n* = 122) and the other for Confirmatory Factor Analysis (CFA, *n* = 115).

### 2.3. Measures

#### 2.3.1. Development of the Simple Qi Deficiency Score

The original version of Qi Deficiency Score was developed by Katsutoshi Terasawa [12,14]. Terasawa’s original criteria combine subjective complaints with objective physical signs. We applied the principle of experiential equivalence, excluding items that patients could assess themselves, e.g., weak pulse, appearance of the tongue, for patient-reported measures. The simple Qi deficiency score comprised eight subjective items (Appendix A Table A1).

#### 2.3.2. Score Refinement

The original Qi Deficiency Score uses a weighting system (full score for distinct symptoms, half for moderate ones). We converted this weighting system into a 4-point Likert scale (“None”, “Mild”, “Moderate”, “Severe”) for clinical use. Scores were weighted according to the importance of the symptom, e.g., “ Body feels tired (now)” is weighted out of 10, while “Loss of appetite” is scored out of 4. ‘None’, ‘Mild’, “Moderate” and ‘Severe’ are assigned 0%, 50%, 75% and 100% of the maximum score, respectively. Details regarding exact point values of each item are provided in Appendix A Table A1. The total possible score ranges from 0 to 56 points, with higher scores revealing more severe Qi deficiency.

#### 2.3.3. English Terminology for Publication

In this study, the original Japanese version of the simple Qi deficiency score was administered to all participants. Multiple researchers translated the items through a consensus discussion to ensure that the English phrasing accurately reflects the original clinical concepts of Qi deficiency in JTM. This translation process was carefully based on English terminology from official textbooks established by the Japan Society for Oriental Medicine [14]. Finally, we ensured that the English phrasing accurately reflects the original clinical concepts (Qi deficiency in JTM).

#### 2.3.4. Reliability and Validity Assessment

Reliability: We calculated Cronbach’s alpha and McDonald’s omega to assess internal consistency [15,16]. Structural validity was evaluated using a two-step approach. First, an exploratory factor analysis (EFA) was conducted (*n* = 122). The Kaiser-Meyer-Orkin (KMO) measure of sampling adequacy and the Bartlett test of sphericity were first checked to verify suitability of the dataset for EFA. A KMO value of at least 0.60 and a significant Bartlett sphericity test were recommended [17]. After exploring the number of factors based on eigenvalues derived from principal component analysis, EFA was performed using the maximum likelihood method. A Promax rotation (an oblique rotation) was applied, assuming that symptoms related to Qi deficiency are correlated.

Subsequently, confirmatory factor analysis (CFA) using structural equation modeling was performed on a separate sample (*n* = 115) to test the fit of a unidimensional (onefactor) model. Model fit was assessed using the comparative fit index (CFI), the Tucker–Lewis index (TLI), the root mean square error of approximation (RMSEA), and the standardized root mean square residual (SRMR). We considered values of CFI 0.90 and RMSEA/SRMR 0.08 as indicative of good fit [17,18,19].

Convergent and discriminant validity: We examined Pearson’s and Spearman’s correlation coefficients between the simple Qi deficiency score and comparator scales. Specifically, the FAS was used to assess convergent validity, whereas the SDS and QOL were used to evaluate discriminant validity.

Fatigue Assessment Scale (FAS): We utilized the Japanese version of the FAS as our primary comparator. This 10-item scale assesses both physical and mental fatigue. Crucially, we have previously validated this specific version for use in Japanese long-COVID patients, where it demonstrated a two-factor structure and high internal consistency (Cronbach’s α = 0.89) [13].

Self-rating Depression Scale (SDS): To distinguish Qi deficiency from depression (discriminant validity) [20,21].

Quality of Life (QOL): Assessed via a linear scale (EQ-5D VAS) to determine the impact of symptoms on general well-being [22].

##### Statistical Analysis

Data were analyzed using Stata/SE 18.0 (StataCorp, College Station, TX, USA).

##### Ethics Approval

For ethical reasons, we employed an opt-out, thereby providing patients with an opportunity to refuse the use of their research data. This study was approved by the Ethics Committee of Okayama University Hospital (No. 2408-041), and the study adhered to the Declaration of Helsinki.

## 3. Results

### 3.1. Participant Profile

This study included 237 patients. A typical demographic profile for long-COVID patients in Japan is shown in Table 1. The mean age was approximately 43 years, and 51% were female.

### 3.2. Reliability

The 8-item simple Qi deficiency score showed a Cronbach’s alpha of 0.64 and a McDonald’s omega of 0.63.

### 3.3. Structural Validity

**Figure A1:**
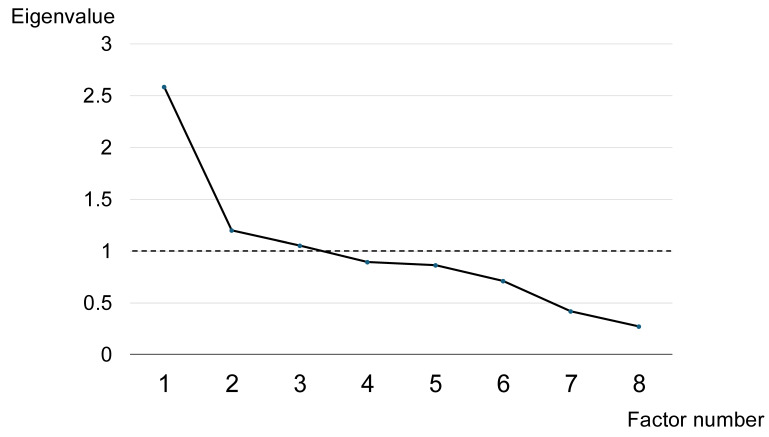
The Scree Plot of the Simple Qi Deficiency Score.

EFA was conducted on the first data set (*n* = 122). The Kaiser-Meyer-Orkin (KMO) measure was 0.696, indicating acceptable sampling adequacy, and Bartlett’s test of sphericity was significant (χ^2^ = 186.57, df = 28, *p* < 0.001), confirming that these data were appropriate for factor analysis. Results of the principal component analysis showed that initial eigenvalues of the first three principal components were 2.58, 1.20 and 1.05, respectively. Scree plots for all items are shown in Appendix A Figure A1. Given the decline in eigenvalues from the first principal component, this indicated that the one-factor model was adequate for this simple Qi deficiency score. EFA was performed using the maximum likelihood method with Promax rotation (Table 2). The two extracted factors explained 47.3% of the cumulative variance. The correlation between the two extracted factors was *r* = 0.29. Although factor extraction was also considered, we decided that a one-factor structure was most appropriate for clinical application, given that ‘Qi deficiency’ has a theoretical basis as a holistic clinical syndrome and that its core symptoms load primarily on the first factor. Consequently, we decided to proceed with validation of a one-factor model in the subsequent confirmatory factor analysis (CFA). Following the EFA (Table 2), we conducted CFA (Table 3) on the second dataset (*n* = 115) to verify the one-factor model. Table 2 displays the standardized factor loadings. The items “Body feels tired (now)” (0.98) and “Tires easily (tendency)” (0.73) exhibited the strongest associations with the latent factor. Related subjective symptoms (“Easy to catch a cold” and “Sleepiness during the day”) showed lower factor loadings. In the confirmatory factor analysis (CFA), the unidimensional model yielded the following fit indices: CFI = 0.871, TLI = 0.820, RMSEA = 0.079, and SRMR = 0.077. Although the RMSEA and SRMR indicated acceptable model fit, the CFI and TLI were slightly below the generally accepted threshold of 0.90. Findings of this study suggest that the model fit is limited. Although a two-factor model was also tested, factor loadings for several items were insufficient (~0.2). Therefore, we think that the utility of a two-factor model is limited. Taking into account the overall clinical nature of ‘Qi deficiency’ and in order to maintain content validity, a one-factor model was adopted.

Criterion-related validity shows that the Simple Qi Deficiency Score had a strong positive correlation with the FAS (*r* = 0.61, ρ = 0.60) (Table 4), which confirms that the score accurately measures the severity of fatigue. The moderate positive correlation with a self-rating depression scale (*r* = 0.55, ρ = 0.55) indicates a relationship between the two conditions. EQ-5D-5L also shows a moderate negative correlation with the Simple Qi Deficiency Score.

## 4. Discussion

This is the first study showing the validity and reliability of the Simple Qi Deficiency Score in the context of post-COVID-19 conditions and long COVID. Our findings demonstrate that this patient-reported outcome is modest for assessing Qi deficiency in clinical practice.

CFA results (CFI = 0.871) support the mild structural validity of the score as a measure of a single construct: Qi deficiency. “General fatigue” and “Tendency to tire easily” were the dominant indicators, aligning with the core definition of Qi deficiency. However, related symptoms such as “Easy to catch a cold” and “Diarrhea tendency” showed lower loadings. While Qi deficiency comprises classic signs in traditional contexts (representing lung and spleen Qi deficiency, respectively) [23], there may be some bias in scoring that reflects these subtypes [23].

The strong correlation with the FAS (*r* = 0.61, ρ = 0.60) provides robust evidence of convergent validity. Since the FAS has been independently validated for this population, its relationship with our score confirms that we are capturing pathological fatigue.The relationship with the SDS (*r* = 0.55, ρ = 0.55) is also noteworthy. Qi deficiency can lead to depressive states (apathy) and depression, as classified according to the JTM. This correlation is not very high; therefore, Qi deficiency remains a clinical entity distinct from major depression.

Cronbach’s α = 0.64 and a McDonald’s ω of 0.63 indicated internal consistency. While a value of 0.70 is preferred [24], values of 0.64 and 0.63 are reasonable, given that “Qi deficiency” is a complex syndrome involving digestion, immunity, and psychological symptoms. Furthermore, patient may suffer from fatigue and weakness without experiencing gastrointestinal distress. Consequently, item-to-item correlations are understandably lower than scales measuring a single, narrow symptom. Cronbach’s α = 0.64 and McDonald’s ω = 0.63 reflect this multidimensionality. Additionally, because the simple Qi deficiency score comprised 8 items, this also contributed to low Cronbach’s α and McDonald’s ω [25].

Fatigue is a common, subjective symptom in general clinical settings. Objective assessment of fatigue has long been challenging in clinical practice [26]. While the FAS provides a useful means of quantifying fatigue using self-reported measures [27], it captures primarily symptom severity and does not necessarily inform treatment selection. This limitation is particularly relevant in JTM, in which even when patients present with similar complaints of fatigue, the appropriate treatment varies depending on the underlying pattern (“Sho”). Therefore, evaluation based solely on symptom severity is insufficient. However, determining such patterns typically requires specialized skills, including pulse measurement and tongue examination, which are not easily implemented by general practitioners and primary care physicians managing long COVID and chronic fatigue [28]. The Simple Qi Deficiency Score addresses this gap by enabling estimation of the Qi deficiency pattern without reliance on specialized techniques.

Moreover, if future research improves the accuracy of the scale and its reliability and validity are sufficiently demonstrated, it may be possible to use it as an indicator of treatment response to Qi-modification formulations such as hochuekkito. This enables visualization of treatment effects that have traditionally relied on subjective clinical judgment. Quantitative evaluation may also extend beyond individual patient care to facilitate prospective and interventional studies, contributing to clinical evidence for JTM. In addition, as a patient-reported outcome measure, the score is well-suited for standardization in different clinical settings and for large-scale data collection, making it a promising tool for multicenter studies and real-world data analyses [29]. Finally, its compatibility with telemedicine further enhances its utility, particularly for long-term follow-up of patients with post-infectious conditions such as long COVID, where access to in-person care may be limited [30].

### Limitations

This study has several limitations. First, it was conducted in a single-center referral population, which may limit the generalizability of our findings to broader primary care or different cultural settings. Participants of this study included only patients at a clinic specializing in long COVID and PCC. This clinic accepts only patients referred from other clinics, and this restriction may have resulted in selection of more severe cases. These patients presented more severe Qi deficiency than the general population. Second, since evaluators included both well-trained and untrained JTM practitioners, this may have resulted in more variable classification, which constitutes an information bias. Third, this study was analyzed using complete-case inclusion. This could potentially generate selection bias if characteristics of patients with missing data were not random. Fourth, goodness-of-fit indices in the CFA (CFI, RMSEA, SRMR and TLI) did not achieve standard optimal thresholds. Although a two-factor model resulted in low factor loadings, the marginal fit of the unidimensional model suggests an inherent psychometric limitation. Qi deficiency is a holistic clinical syndrome, and forcing these diverse, systemic symptoms into a single mathematical dimension naturally reduces the statistical fit. Therefore, while this brief score maintains high content validity and is very useful for rapid clinical screening, its structural validity is only partially supported, representing an oversimplification of the complex Qi deficiency condition. Future longitudinal studies will need to determine responsiveness, test–retest reliability, optimal diagnostic thresholds, and the minimal clinically important difference for this score. Additionally, multicenter cohort studies are required to validate its external applicability and confirm its broader clinical utility. A similar concept exists in traditional Chinese medicine as Qi deficiency [31], and it may be applicable there as well. The clinical use of the simple Qi deficiency score may gain widespread acceptance not only domestically, but also internationally.

## 5. Conclusions

The simple Qi deficiency score may be useful for evaluating Qi deficiewncy in patients with post-COVID-19 condition. The simple numerical score can express complex traditional diagnostic criteria as patient-reported outcomes.

## Figures and Tables

**Table 1:** Participant characteristics (N = 237).

	N (%)
**Age**	
18–29 years	47 (19.8)
30–39 years	50 (21.1)
40–49 years	63 (26.6)
50–59 years	54 (22.8)
60–69 years	15 (6.3)
Over 70 years	8 (3.4)
**Gender**	
Female	121 (51.0)
Male	116 (49.0)
**Acute phase status**	
Admission	46 (19.4)
Home care or care at accommodation facilities	191 (80.1)
**COVID-19 vaccination**	
0 dose	90 (38.0)
1 dose	9 (3.8)
2 doses	74 (31.2)
3 doses	45 (19.0)
4 doses	12 (5.1)
5 doses	4 (1.7)
6 doses	2 (0.8)
7 doses	1 (0.4)

**Table 2:** Results of exploratory factor analysis of the simple Qi deficiency score (N  =  122).

Items of the Simple Qi Deficiency Score	Factor Loading		
	One-Factor	Two-Factor	
	Factor 1	Factor 1	Factor 2
1. Body feels tired (now)	0.8554	0.9814	−0.1246
2. Lack of energy	0.6983	0.5706	0.3228
3. Tires easily (tendency)	0.7928	0.7312	0.0443
4. Sleepiness during the day	0.2028	0.1047	0.2167
5. Loss of appetite	0.2928	0.1594	0.3873
6. Easy to catch a cold	0.1564	0.0229	0.2725
7. Easily surprised (easily startled)	0.1244	−0.0784	0.5283
8. Diarrhea tendency	0.2204	0.1369	0.2164

**Table 3:** Confirmatory factor analysis of the Simple Qi Deficiency Score (N  =  115).

	CFI	RMSEA	SRMR	TLI
Model (one-factor model)	0.871	0.0792	0.0772	0.820

**Table 4:** Criterion-Related Validity.

	Correlation	
	Peason’s *r*	Spearman’s ρ
FAS (N = 235)	0.6065	0.5980
SDS (N = 231)	0.5529	0.5465
QOL (EQ-5D-5L) (N = 228)	−0.4172	−0.4294
QOL (VAS) (N = 228)	−0.3125	−0.3247

## Data Availability

All data may be available when inquiries can be directed to the corresponding author upon request.

## Abbreviations

Comparative fit index (CFI); confirmatory factor analysis (CFA); coronavirus disease 2019 (COVID-19); COVID-19 aftercare clinic (CAC); exploratory factor analysis (EFA); fatigue assessment scale (FAS); Japanese Traditional (Kampo) Medicine (JTM); post-COVID-19 condition (PCC); quality of life (QOL); root mean square error of approximation (RMSEA); self-rating depression scale (SDS); standardized root mean square residual (SRMR); Tucker–Lewis index (TLI).

## Appendix A

Figure legends:

Scree plot of eigenvalues from the principal component analysis. The plot displays the initial eigenvalues for each factor. While the first three components show eigenvalues greater than 1.0 (Kaiser criterion), the first component predominantly accounts for the variance, supporting the extraction of a single, overarching factor for the simple Qi deficiency score.

**Table A1:** The Simple Qi Deficiency Score [14].

	Items	None	Mild	Moderate	Severe
1	Body feels tired (now)	0	5	7.5	10
2	Lack of energy	0	5	7.5	10
3	Tires easily (tendency)	0	5	7.5	10
4	Sleepiness during the day	0	3	4.5	6
5	Loss of appetite	0	2	3	4
6	Easy to catch a cold	0	4	6	8
7	Easily surprised (easily startled)	0	2	3	4
8	Diarrhea tendency	0	2	3	4

**Table A2:** Original version of the Qi Deficiency Score (Japanese).

気虚スコア (Qi Deficiency Score)	
項目 (Items)	最大得点 (Maximum Score)
身体がだるい (Body feels tired (now))	10
気力がない (Lack of energy)	10
疲れやすい (Tires easily (tendency))	10
日中の眠気 (Sleepiness during the day)	6
食欲不振 (Loss of appetite)	4
風邪をひきやすい (Easy to catch a cold)	8
物事に驚きやすい (Easily surprised (easily startled))	4
眼光 ・音声に力がない (Lack of power in eyes and voice)	6
舌が淡白紅 ・腫大 (Tongue pale or swollen (sometimes teeth marked))	8
脈が弱い (Weak pulse)	8
腹力が軟弱 (Weak abdominal strength)	8
臓器のアトニー症状 (Visceral atonic signs)	10
小腹不仁 (Weakness in the lower abdominal region)	6
下痢傾向 (Diarrhea tendency)	4

A total score of ≥30 is classified as Qi deficiency. A full score is awarded for items in which the condition is clearly evident, and half the score is awarded for items in which the condition is mild.

From the Table: Diagnostic Criteria for Qi Deficiency [12].
